# Internal fixation versus total hip arthroplasty for displaced femoral neck fractures in patients aged 60 to 80 years: Patient-reported outcomes and complications

**DOI:** 10.1371/journal.pone.0323106

**Published:** 2025-05-08

**Authors:** Ryo Mitsutake, Hiromasa Tanino, Go Sato, Hiroshi Ito

**Affiliations:** 1 Department of Orthopaedic Surgery, Asahikawa Medical University, Asahikawa, Hokkaido, Japan; 2 Department of Orthopaedic Surgery, Hokkaido Social Welfare Association Furano Hospital, Furano, Hokkaido, Japan; University Hospital Zurich, SWITZERLAND

## Abstract

**Background:**

The treatment of displaced femoral neck fractures in patients aged 60–80 years old remains controversial. Arthroplasty has been reported to have lower complication rates than internal fixation (IF). However, less is known about the outcomes as perceived by the patient. The aim of the present study (the cross-sectional study) was to evaluate the patient-reported outcome measures (PROMs) of patients aged 60–80 years old with femoral neck fractures treated with IF or total hip arthroplasty (THA).

**Methods:**

We investigated 92 patients affected by displaced femoral neck fractures who were treated between January 2015 and September 2022. Forty-eight patients were treated with IF, and 44 patients with THA. The outcomes were Harris Hip Score (HHS), Hip Disability and Osteoarthritis Outcome Score (HOOS), Japanese Orthopaedic Association Hip-Disease Evaluation Questionnaire (JHEQ), visual analogue scale (VAS) for pain, and VAS for patient satisfaction at 12 months postoperatively. Complications and reoperations were continuously monitored.

**Results:**

The mean patient age was 68.1 ± 6.6 years. HHS, all dimensions of the HOOS and JHEQ scores, VAS for pain, and VAS for patient satisfaction at 12 months were significantly superior in the THA group compared to the IF group. All outcome measures were superior in the THA group, with mean differences exceeding their respective minimal clinically important differences or minimal detectable changes at 12 months. The rate of major reoperations was significantly higher in the IF group (14.5%) than the THA group (2.2%).

**Conclusion:**

We found that patients aged 60–80 years old who underwent THA for displaced femoral neck fractures experienced better outcomes, including PROMs, than those who underwent IF. Furthermore, THA resulted in fewer reoperations than IF.

## Introduction

Elderly patients with displaced femoral neck fractures are usually operated with arthroplasty due to lower complication rates and better functional outcomes compared to internal fixation (IF) [1–6]. Age, comorbidities, patient independence, and potential surgical complications must be considered when choosing between total hip arthroplasty (THA) and hemiarthroplasty. Previous studies have suggested that THA results in better patient-reported outcome measures (PROMs) than hemiarthroplasty in elderly patients [7,8]. However, for patients aged 60–80 years old, the decision between IF and arthroplasty remains controversial [9–13]. Most femoral neck fractures in patients aged 60–80 years old occur as a result of low-energy trauma; moreover, these patients have symptomatic comorbidities that may increase the risk of failed IF [14–16]. For patients younger than 60 years old, preservation of the femoral head and IF are the main concerns [17–19]. IF is less invasive than arthroplasty but has a higher reoperation rate [20]. Furthermore, the risk of complications is even higher in salvage surgery after failed IF [21–23]. The commonly used endpoints for reoperation represent potential consequences of unsuccessful treatment. However, PROMs for femoral neck fractures have not been fully evaluated [10–13,24,25], and there are few studies on disease-specific PROMs of femoral neck fractures [11]. Therefore, we investigated outcomes after displaced femoral neck fractures in patients between 60 and 80 years old treated by IF or THA.

## Materials and methods

### Study population

**Fig 1:**
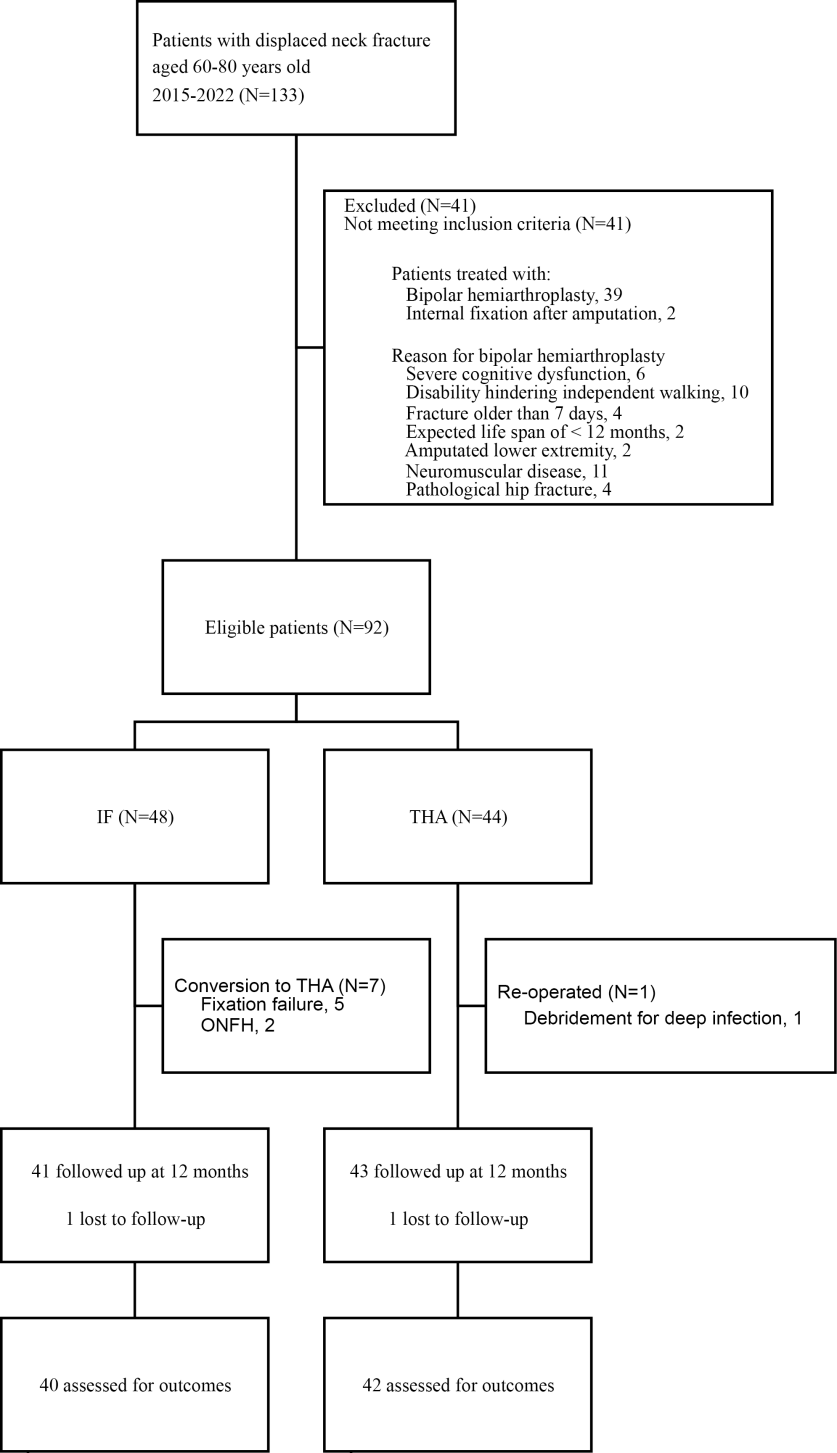
Flow chart of patients included in the cross-sectional study.

The study protocol was approved by our hospital’s Institutional Review Board (AMU 24004). All participants provided written informed consent. We accessed the data for research purposes on May 17, 2024. This retrospective study included 133 patients treated for femoral neck fractures between January 2015 and September 2022. This study was conducted at two hospitals: one is a university hospital that has more THA cases, and the other is a local hospital that has more IF cases. Inclusion criteria for this study were acute displaced femoral neck fractures (Grade Ⅲ or Ⅳ using Garden classification [26]), age between 60 and 80 years old, normal cognitive function (mini-mental test score of > 24 [27]), and independent ambulation before the injury. The minimum follow-up was one year. Exclusion criteria were a femoral neck fracture older than 7 days, concomitant pelvic or lower extremity fracture, an expected life span of < 12 months as judged by the surgeon, an American Society of Anesthesiologists (ASA) grade of 4 or 5 [28], an amputated lower extremity, neuromuscular diseases, or pathological hip fracture. Patients were allocated into the IF or THA groups depending on treatment (48 and 44 patients, respectively, Fig 1).

Patients were prioritized for surgery no later than 48 hours after admission. IF was carried out with the patient on a fracture table. The fractures were reduced by closed manipulation using an image intensifier. After the reduction, IF was achieved using Pinloc [29,30]. Pinloc was developed to provide mechanical stability for rotational displacement during fracture healing. Pinloc consists of three cylindrical parallel pins with hooks, which are connected through a fixed angle interlocking plate. Radiographic analysis was performed for all patients using follow-up radiographs. Reduction quality was measured according to the Garden classification and was classified as acceptable (within the range of 155–180° in both anteroposterior and lateral radiographs), and borderline to unacceptable (< 155 or > 180° in either view). Loss of fracture reduction was defined as greater than 20° change in angulation and/or greater than 5 mm translation in AP or lateral views.

THA was carried out using the posterolateral approach with the patient in the lateral decubitus position. All femoral components were cemented. Twelve patients received 4-U (Nakashima Medical Co., Japan [31,32]) and 32 CMK Original Concept Stem (Zimmer Biomet, Warsaw, IN, USA). All acetabular components were uncemented. There were eight 4-U cups (Nakashima Medical Co., Japan), four GS cups (Nakashima Medical Co., Japan), 20 Continuum cups (Zimmer Biomet, Warsaw, IN, USA), and 12 G7 cups (Zimmer Biomet, Warsaw, IN, USA). A prosthetic femoral head diameter of 32 mm or dual mobility cup was utilized. Surgeons used a 32 mm head in the periods from January 2015 to February 2017 and from October 2018 to September 2022, whereas they used dual mobility cup from March 2017 to September 2018 in most of the cases. All patients received perioperative antibiotic prophylaxis and anticoagulation prophylaxis. In both groups, early mobilization and full weight-bearing were allowed.

The patients were interviewed about their mobility, activities of daily living, living conditions, and comorbidity during the last week before the fracture. They attended clinical and radiological reviews at 12 months. We assessed hip function according to Harris Hip Score (HHS) and assessed PROMs according to the Hip Disability and Osteoarthritis Outcome Score (HOOS), and Japanese Orthopaedic Association Hip-Disease Evaluation Questionnaire (JHEQ) [33–35]. HHS is a surgeon-administered measurement for assessing hip function. HHS includes sections on pain (0–44 points), function (0–47 points), absence or presence of deformity (0–4 points), and passive range of motion (0–5 points), and is scored from 0 (worst) to 100 (best). Postoperative scores greater than 70 are considered fair, scores greater than 80 are considered good, and scores greater than 90 are considered excellent. There are two main types of PROMs that are distinguished by different levels of focus. Generic instruments are designed to provide a measure of general health for any health state, regardless of the presence or absence of illness, disability, or specific symptoms; thus, generic PROMs describe a patient’s global health status and are comparable across different conditions. Disease-specific PROMs focus on specific symptoms, diseases, organs, body regions, or body functions. Disease-specific PROMs may also be specifically designed to measure the effect of a specific intervention or treatment. The most commonly used disease-specific PROM is HOOS, followed by JHEQ. HOOS is based on five subscales that measure pain, symptoms, function in activities of daily living, function in sport and recreation, and quality of life. Scores for each subscale are scored from 0 (worst) to 100 (best). Scores for each subscale greater than 70 are considered fair, scores greater than 80 are considered good, and scores greater than 90 are considered excellent. JHEQ is a self-administered questionnaire that includes questions related to common Asian-lifestyle situations involving movement such as using a Japanese-style toilet or getting up from the floor. The JHEQ is based on three subscales that measure pain, movement, and mental wellbeing. Scores for each subscale are scored from 0 (worst) to 28 (best). Minimal clinically important differences (MCIDs) and minimal detectable changes (MDCs) were defined according to clinical practice and previously published data [11,36–40]. The MCID for the HHS was 10. The MCIDs of HOOS total, pain, symptoms, activities of daily living, sport and recreation, and quality of life were 10, 10, 11, 10, 15, and 13, respectively. The MDCs of JHEQ pain, movement, and mental wellbeing were 2.6, 2.0, and 2.9, respectively. We also assessed pain and patient satisfaction using visual analogue scales (VAS). Activities of daily living were assessed using University of California-Los Angeles (UCLA) activity scale score [41]. Living conditions were categorized as independent or institutionalized. Comorbidities were reported using ASA classification [28]. VAS for pain and VAS for patient satisfaction were measured on a 100-point scale, with 0 indicating favorable results (no pain and the highest possible satisfaction) and 100 indicating unfavorable results (unbearable pain and the lowest possible satisfaction). The MCIDs for VAS for pain and VAS for satisfaction were 10 and 10, respectively. Complications and reoperations were recorded. Reoperations were categorized as major or minor. Minor reoperations were defined as the removal of screws only, an open reduction of a dislocated THA, or debridement for superficial infection.

### Statistical analysis

Data are reported using descriptive statistics, including mean, standard deviation, and range values. For continuous variables, normality was assessed using Shapiro-Wilk test, and statistical analysis was performed using paired t-test and non-parametric Mann-Whitney U test. Chi-square test was used for analysis of categorical data. P-values of < 0.05 were considered significant. All statistical analyses were performed using SPSS version 25 (SPSS Inc., Chicago, IL).

## Results

The mean patient age was 68.1 ± 6.6 years old. Mean ages were 68.2 and 68.0 years old for the IF and THA groups, respectively. No significant differences were found in sex, mean age, BMI, ASA classification, Charlson comorbidity index score, UCLA activity scale score, current smokers, alcohol abuse, residence, mechanism of injury, and ability to walk with or without walking aids between the two groups (Table 1). No significant difference was detected in terms of the demographic data following exclusion between the two hospitals.

**Table 1:** Demographic data of subjects.

Parameters	IF (N = 48)	THA (N = 44)	p-value
Sex^*^			0.148
Male	15 (31.2)	8 (18.1)	
Female	33 (68.7)	36 (81.8)	
Age (years)^†^	68.2 ± 7.0 (60-80)	68.0 ± 6.1 (60-79)	0.871
BMI (kg/m^2^)^†^	20.9 ± 2.8 (14.8-31.2)	21.4 ± 3.7 (13.2-31.1)	0.533
ASA classification^*^			0.458
1-2	41 (85.4)	35 (79.5)	
3	7 (14.5)	9 (20.4)	
Charlson comorbidity index score^†^	3.6 ± 1.2 (2-6)	3.8 ± 1.1 (2-6)	0.549
UCLA activity scale score^†^	6.4 ± 1.3 (4-9)	5.9 ± 1.3 (3-9)	0.070
Current smokers^*^	13 (27.0)	10 (22.7)	0.630
Alcohol abuse^*^	5 (10.4)	5 (11.3)	0.573
Residence^*^			0.922
Living at home	40 (83.3)	37 (84.0)	
Institution	8 (16.6)	7 (15.9)	
Fall from standing height^*^			0.231
Indoors	17 (35.4)	21 (47.7)	
Outdoors	31 (64.5)	23 (52.2)	
Mobility^*^			0.573
No walking aids	43 (89.5)	39 (88.6)	
Stick	5 (10.4)	5 (11.3)	

* The number of patients with the percentage in parentheses;

† The values are given as the mean and standard deviation with the range in parentheses

IF: Internal fixation; THA: Total hip arthroplasty; BMI: Body mass index; ASA: American Society of Anesthesiologists; UCLA: University of California-Los Angeles

Fracture reduction was considered to be acceptable in all 48 patients of the IF group. The mean surgical time for the IF group (46.8 min) was significantly lower than the THA group (80.7 min) (p < 0.001). The mean intraoperative blood loss for the IF group (15.9 mL) was significantly lower than the THA group (276.2 mL). The need for blood transfusion for the IF group was significantly lower (0 hips) than the THA group (14 hips). There were no differences in the rates of postoperative complications between the groups (Table 2).

**Table 2:** Surgical details.

Parameters	IF (N = 48)	THA (N = 44)	p-value
Surgical time (min)^†^	46.8 ± 17.2 (25-114)	80.7 ± 15.8 (50-116)	<0.001
Intraoperative blood loss (mL)^†^	15.9 ± 34.9 (0-200)	276.2 ± 121.8 (66-494)	<0.001
Need for blood transfusion^*^	0 (0)	14 (31.8)	<0.001
Postoperative complication^*^	1 (2.0)	2 (4.5)	0.467
Urinary tract infection	0 (0)	1 (2.2)	
Myocardial infarction	0 (0)	0 (0)	
Pneumonia	1 (2.0)	0 (0)	
Deep vein thrombosis	0 (0)	0 (0)	
Pulmonary embolism	0 (0)	0 (0)	
Dislocation after THA		1 (2.2)	

† The values are given as the mean and standard deviation with the range in parentheses;

* The number of patients with the percentage in parentheses

IF: Internal fixation; THA: Total hip arthroplasty

**Fig 2:**
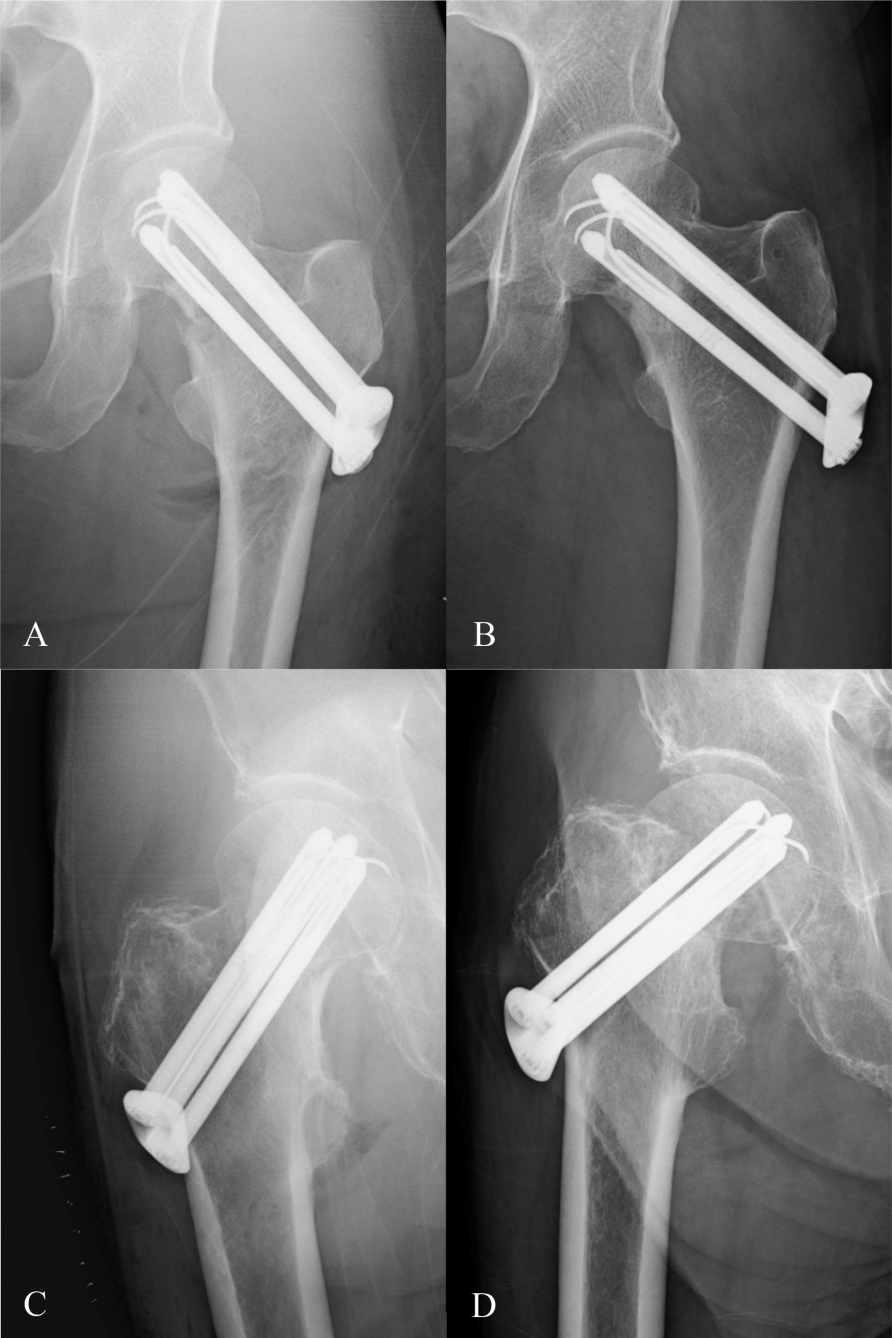
Two typical scenarios of IF. Immediate postoperative X-ray of a 60-year-old woman with an acute displaced femoral neck fracture treated with internal fixation using Pinloc (A) and the corresponding 12-month follow-up X-ray (B), showing fracture healing. Immediate postoperative X-ray of an 80-year-old man with an acute displaced femoral neck fracture treated with internal fixation using Pinloc (C) and the corresponding 6-month follow-up X-ray (D), showing the non-union of femoral neck fracture.

The rate of reoperation was significantly higher in the IF group (29.1% [14 of 48], with 14.5% having a subsequent arthroplasty and 14.5% screw removal) than the THA group (2.2% [1 of 44] having soft tissue debridement for deep infection) (p < 0.001). Of these, the rate of major reoperations was significantly higher in the IF group (14.5% [7 of 48] having a subsequent arthroplasty) than the THA group (2.2% [1 of 44] having soft tissue debridement for deep infection) (p = 0.039) (Table 3). Fig 2 illustrates two typical scenarios of IF.

**Table 3:** Reoperations.

Parameters	IF (N = 48)	THA (N = 44)	p-value
Reoperation^*^	14 (29.1)	1 (2.2)	<0.001
Major reoperation^*^	7 (14.5)	1 (2.2)	0.039
Nonunion	5 (10.4)		
Osteonecrosis	2 (4.1)		
Deep infection	0 (0)	1 (2.2)	
Periprosthetic fracture		0 (0)	
Mechanical loosening		0 (0)	
Recurrent dislocation		0 (0)	
Minor reoperation^*^	7 (14.5)	0 (0)	0.008
Screw removal	7 (14.5)		
Open reduction		0 (0)	
Superficial infection	0 (0)	0 (0)	

* The number of patients with the percentage in parentheses

IF: Internal fixation; THA: Total hip arthroplasty

All patients included in this study were routinely followed up. The average length of follow-up was 4.1 ± 2.5 years (range, 0.5–8.0 years). Follow up radiography was performed at final follow up evaluation. In the THA group, there were no signs of radiological loosening of the components in any of the patients at the final follow up and no patients in the THA group underwent a major reoperation from 12 months to final follow up. In the IF group, three patients suffered osteonecrosis and underwent a major reoperation from 12 months to final follow up.

### Outcome measures

Seven patients (14.5%) in the IF group and one patient (2.2%) in the THA group underwent a major reoperation during the 12-month period. These patients, who likely had relatively worse functional outcomes and PROMs before reoperation, were not followed up by questionnaire at 12 months (Fig 1). The HHS at 12 months was higher in the THA group (87.6 ± 8.5) than the IF group (75.4 ± 18.9) (Table 4). For PROMs, the THA group had better results than the IF group, including significantly better scores in all dimensions of the HOOS and JHEQ at 12 months (Table 4). VAS for pain and VAS for patient satisfaction at 12 months were significantly superior in the THA group compared to the IF group (Table 4). All outcome measures were superior in the THA group, with mean differences exceeding their respective MCIDs or MDCs at 12 months.

**Table 4:** Outcomes.

	IF (N = 40)	THA (N = 42)	IF Versus THA	
Parameters	Mean ± SD (range)	Mean ± SD (range)	Mean Diff. (95% CI)	p-value
HHS	75.4 ± 18.9 (32-96)	87.6 ± 8.5 (67-100)	-12.1 (-18.7 to -5.6)	<0.001
HOOS	69.9 ± 17.2 (28.1-86.9)	85.2 ± 9.3 (57.5-100)	-15.3 (-21.4 to -9.1)	<0.001
HOOS pain	74.5 ± 14.1 (37.5-92.5)	89.7 ± 9.6 (60-100)	-15.2 (-20.7 to -9.7)	<0.001
HOOS symptoms	70.2 ± 13.1 (25-85)	83.4 ± 9.5 (60-100)	-13.2 (-18.2 to -8.1)	<0.001
HOOS activities of daily living	73.9 ± 18.9 (20.5-92.6)	86.8 ± 9.2 (60.2-100)	-12.8 (-19.5 to -6.1)	<0.001
HOOS sport and recreation	49.5 ± 32.5 (0-87.5)	75.8 ± 12.6 (50-100)	-26.3 (-37.4 to -15.3)	<0.001
HOOS quality of life	61.4 ± 20.7 (0-81.2)	78.4 ± 12.5 (43.7-100)	-17.0 (-24.6 to -9.3)	<0.001
JHEQ pain	20.3 ± 4.5 (6-24)	25.2 ± 2.5 (21-28)	-4.9 (-6.5 to -3.2)	<0.001
JHEQ movement	14.8 ± 7.6 (0-25)	18.2 ± 4.7 (11-28)	-3.3 (-6.1 to -0.5)	0.021
JHEQ mental	20.3 ± 6.2 (6-28)	23.3 ± 4.0 (15-28)	-3.0 (-5.3 to -0.6)	0.012
VAS for pain	33.6 ± 31.3 (5-93)	16.2 ± 8.8 (0-41)	17.4 (7.0 to 27.8)	0.001
VAS for patient satisfaction	32.8 ± 31.2 (5-91)	15.4 ± 8.7 (0-40)	17.3 (7.0 to 27.6)	0.001

IF: Internal fixation; THA: Total hip arthroplasty; HHS: Harris Hip Score; HOOS: Hip Disability and Osteoarthritis Outcome Score; JHEQ: Japanese Orthopaedic Association Hip-Disease Evaluation Questionnaire; VAS: Visual analogue scale

## Discussion

We investigated the outcomes after displaced femoral neck fractures in patients aged between 60 and 80 years old treated by IF or THA. In our study, all outcome measures were superior in the THA group, with mean differences exceeding their respective MCIDs or MDCs at 12 months. THA resulted in less pain, better patient satisfaction, better quality of life, and fewer reoperations than IF.

The treatment of displaced femoral neck fractures is controversial [2,9–13,17,18,24,25,42–50]. Treatment is based on the patient’s age, functional demand, and individual risk profile. Most studies on this subject focus on complications and the need for further surgery rather than function of the hip; thus, the PROMs of femoral neck fractures have not been evaluated fully [10–13,24,25]. Especially, there are few studies on disease-specific PROMs of femoral neck fractures [11]. Previous studies have reported better results after THA compared to IF in terms of overall surgeon-administered measurements for assessing hip function, function of abductor muscles, and independent ambulation without walking aids [9,42,43,48].

In the present study, seven patients (14.5%) in the IF group underwent a major reoperation during the 12 months. This is equal to or better than those in most previous studies [10,11,47–50]. The Pinloc was developed to provide better stability and, therefore, to decrease the reoperation rate. These patients, who likely had relatively worse functional outcomes and PROMs before reoperation, were not followed up by questionnaire at 12 months; thus, this study likely overestimated the effects of treatment with IF. When considering only patients with an uneventful postoperative course, functional outcomes, and PROMs at 12 months after THA were better. According to the current best practice, fracture reduction and screw position were optimal in the present study; however, the configuration and the numbers of screws utilized may have affected the rate of reoperation following IF. Furthermore, surgeon experience and the quality of reduction may affect fracture healing and failure [51,52]. The mean surgical time for the IF group (46.8 min) is comparable with that in previous studies [9,11].

Although surgical time and operative blood loss were significantly lower in the IF group, THA resulted in less pain, better patient satisfaction, better quality of life, and fewer reoperations than IF. This supports previous studies with displaced femoral neck fractures [10,11,13,24,25]. On the other hand, another study did not show any difference in PROMs between the IF and THA groups [12].

To the best of our knowledge, no previous clinical studies that examined the disease-specific PROMs of femoral neck fractures using JHEQ have been reported. In a study of femoral neck fractures, Bartels et al. reported that a THA group had significantly better disease-specific PROMs, including HOOS and Oxford Hip Score, than an IF group [11]. In the present study, the THA group had better results than the IF group for disease-specific PROMs, including significantly better scores in all dimensions of HOOS and JHEQ scores, which supports the findings of Bartels et al. The disease-specific PROMs were constructed with items that sensitively detect health status specific to a certain disease. JHEQ includes disease-specific PROMs that are associated with common Asian-lifestyle situations requiring movements that use deep flexion [35].

Deciding whether to perform IF or THA for the treatment of patients aged 60–80 years old remains controversial and there are few previous studies [9–13]. In this age group, the optimal treatment should be individualized depending on the fracture pattern, preoperative ambulation, level of independence, disability, and general health status of the patient. Our study found better functional outcomes after THA, which supports previous studies with displaced femoral neck fractures in this age group [9–11]. On the other hand, other study did not show any difference in PROMs between the IF and THA groups in patients aged 60–80 years old [12,13]. The majority of the patients included were relatively young for this category, with a mean age of 68.1 years. This may mean that our findings were most valid for patients ≤ 75 years old.

The present study had several limitations. First, the follow-up period was limited to 1 year. The 1 year follow-up may have been too short to detect late complications. A longer follow-up could naturally highlight the risk of revision arthroplasty in patients who undergo primary IF. Although IF patients may experience late complications and a high-risk rate of revision arthroplasty, previous long-term follow-up studies identified few or no late revisions of primary THA [48,50]. Second, we did not perform a randomized controlled trial. However, we found no significant difference in baseline characteristics between the patient groups. Third, hemiarthroplasty was not taken into account in this study. Hemiarthroplasty has been established as the treatment of choice for displaced femoral neck fractures [4,5]; however, it has also been shown to have an unacceptably high risk of pain and revision compared with THA when used to treat degenerative osteoarthritis or osteonecrosis of the femoral neck [6,53,54]. This has led to concern that long-term survivors after hip fracture may experience late problems following hemiarthroplasty; however, the present study found no evidence to support this. Finally, the higher complication rate in the IF group was associated with better PROMs in patients with an uneventful postoperative course, which may impact comparisons between the groups. Therefore, this study likely overestimated the effects of treatment with IF.

With less pain, higher satisfaction, higher quality of life, and fewer reoperations, the patients treated with THA had better results than the patients treated with IF at 12 months. All outcome measures were superior in the THA group, with mean differences exceeding their respective MCIDs or MDCs at 12 months. THA for treatment of displaced femoral neck fractures significantly reduced the risk of reoperation at the cost of higher surgical time and blood loss compared with IF. Future trials comparing IF and THA in patients should include large cohorts and focus on long-term results.

## Conclusions

Patients aged between 60 and 80 years old who underwent THA for displaced femoral neck fractures experienced better outcomes than those who underwent IF.

## Supporting information

S1 Data All relevant data. (XLSX)

S1 Checklist STROBE Statement—Checklist of items that should be included in reports of *cross-sectional studies.* (DOCX)
